# Otogenic Lateral and Transverse Sinovenous Thrombosis in a Child: A Case Report

**DOI:** 10.7759/cureus.84045

**Published:** 2025-05-13

**Authors:** Astrid Rosero-Castillo, Diana Almendariz-Ramos, Jose Treviño-González

**Affiliations:** 1 Otolaryngology - Head and Neck Surgery, Hospital Universitario Dr. José Eleuterio González, Universidad Autonoma De Nuevo Leon, Monterrey, MEX

**Keywords:** acute media otomastoiditis, anticoagulants, child, intracranial thrombosis, sinus thrombosis

## Abstract

Acute otitis media (AOM) is common in children, but intracranial complications such as meningitis, cerebral abscess, and cerebral venous sinus thrombosis are rare and can be potentially life-threatening. This case describes a three-year-old boy who presented to the emergency room following one week of coryza, fever, cough, malaise, and otalgia, along with three days of severe headache and vomiting. Physical examination revealed hyperemia and bulging of both tympanic membranes on otoscopy, along with an elevated erythrocyte sedimentation rate and elevated levels of leukocytes, C-reactive protein, and D-dimer. Cranial computed tomography scan revealed soft tissue density occupying the mastoid cells bilaterally and enlargement of the right transverse and sigmoid venous sinuses; magnetic resonance imaging of the brain revealed thrombophlebitis in the right internal jugular vein and thrombosis in the transverse and sigmoid sinuses. The diagnosis was bilateral AOM with otogenic lateral right sinus thrombosis, and the patient had also developed bilateral sixth cranial nerve paresis and cerebellitis. He made a full recovery without any sequelae, following treatment that included the placement of tympanostomy tubes in both ears, a 21-day course of systemic antibiotics, and two weeks of anticoagulation therapy with low molecular-weight heparin. Follow-up showed clinical improvement and normalization of inflammatory markers; the radiological images showed complete resolution of thrombosis. Since otogenic lateral sinus thrombosis symptoms can mimic uncomplicated AOM, maintaining a high index of suspicion is essential in preventing fatal outcomes and ensuring favorable recovery in pediatric patients.

## Introduction

Acute otitis media (AOM) is common in childhood. By the age of 3, at least 60% of children have experienced at least one episode of AOM, and approximately 24% have had three or more episodes within this period. The main complication is acute mastoiditis (AM), with variable incidence between 0.3% and 1% in the era of antibiotics; however, since the introduction of the pneumococcal conjugate vaccine, changes in the microbiological landscape and the epidemiology due to the reduced incidence of Streptococcus pneumoniae [1,2]. Meanwhile, intracranial complications from AOM are seen in 0.1% to 0.3% of patients. Very rarely, lateral sinovenous thrombosis is observed in 2.7% of the cases [1,3]; it is a life-threatening condition, with mortality rates from 5% to 10%. Most clinical findings with lateral sinovenous thrombosis resemble those seen in children with AOM, and, due to its subtle clinical presentation, suspicion is essential for a prompt diagnosis and appropriate management [4,5].

In this case study, we report a three-year-old boy with thrombosis of the transverse sinus secondary to bilateral AOM, who responded to antibiotic and anticoagulation treatment. We discussed the clinical presentation, diagnostic approach, treatment strategy, and follow-up.

## Case presentation

A three-year-old boy from an urban area, with a complete immunization schedule for his age and healthcare worker parents, was evaluated in the pediatric emergency department of a tertiary care hospital for intense headache, vomiting, and two episodes of horizontal nystagmus observed by his parents. He had a history of primary hyperthyroidism and allergic rhinitis, without previous acute or chronic otitis. His symptoms were preceded by one week of coryza, fever, cough, malaise, and bilateral otalgia, for which he received amoxicillin/clavulanic acid; however, the exact dosage was unknown. At admission, his vital signs were as follows: blood pressure 90/60 mmHg, respiratory rate 22 breaths per minute, heart rate 120 beats per minute, temperature 36.5 °C, blood oxygen saturation 98% in ambient air, weight 14 kg, and height 94 cm. On physical examination, he was alert and crying, without at-rest or evoked nystagmus and with facial and eye movements appearing conserved. Negative signs of meningeal irritation, there was an increase in volume, erythema, or significant pain on palpation in the mastoid regions bilaterally. He had normal bilateral auricles; otoscopy revealed hyperemic and bulging tympanic membranes bilaterally, grade 3 tonsils without exudate in the oropharynx, a non-linear gait, and inability to complete vestibular assessment due to irritability. The laboratory test results are shown in Table 1.

**Table 1:** Laboratory findings. *C-reactive protein. **Erythrocyte sedimentation rate. INR, international normalized ratio

Variable	Values					Range
	ED admission	2nd day	3rd day	7th day	12th day	
Hemoglobin (g/dL)	11.3		11.3		11.5	12.2-18.1
Hematocrit (g/dL)	34.5		33.5		34.9	
Leukocytes	10.2		14.3		9.46	4.0 -11.0
Neutrophils (K/uL)	6.1		8.67		2.66	2.0 -6.90
Lymphocytes (K/uL)	3.1		4.15		5.47	0.60-3.40
Platelets (K/uL)	471		572		652	142.0 -24.0
Activated partial thromboplastin time (seconds)	32				36.3	28.5-36.8
Prothrombin time (seconds)	12.5				11.3	9.89-12.6
INR	1.13				1.03	
CRP* (mg/dL)	5.3		1.8		0.5	0-1.0
ESR** (mm/hour)	48		38		28	0-9
D-dimer (ng/dL)		991		959		0-243.0

**Figure 1:**
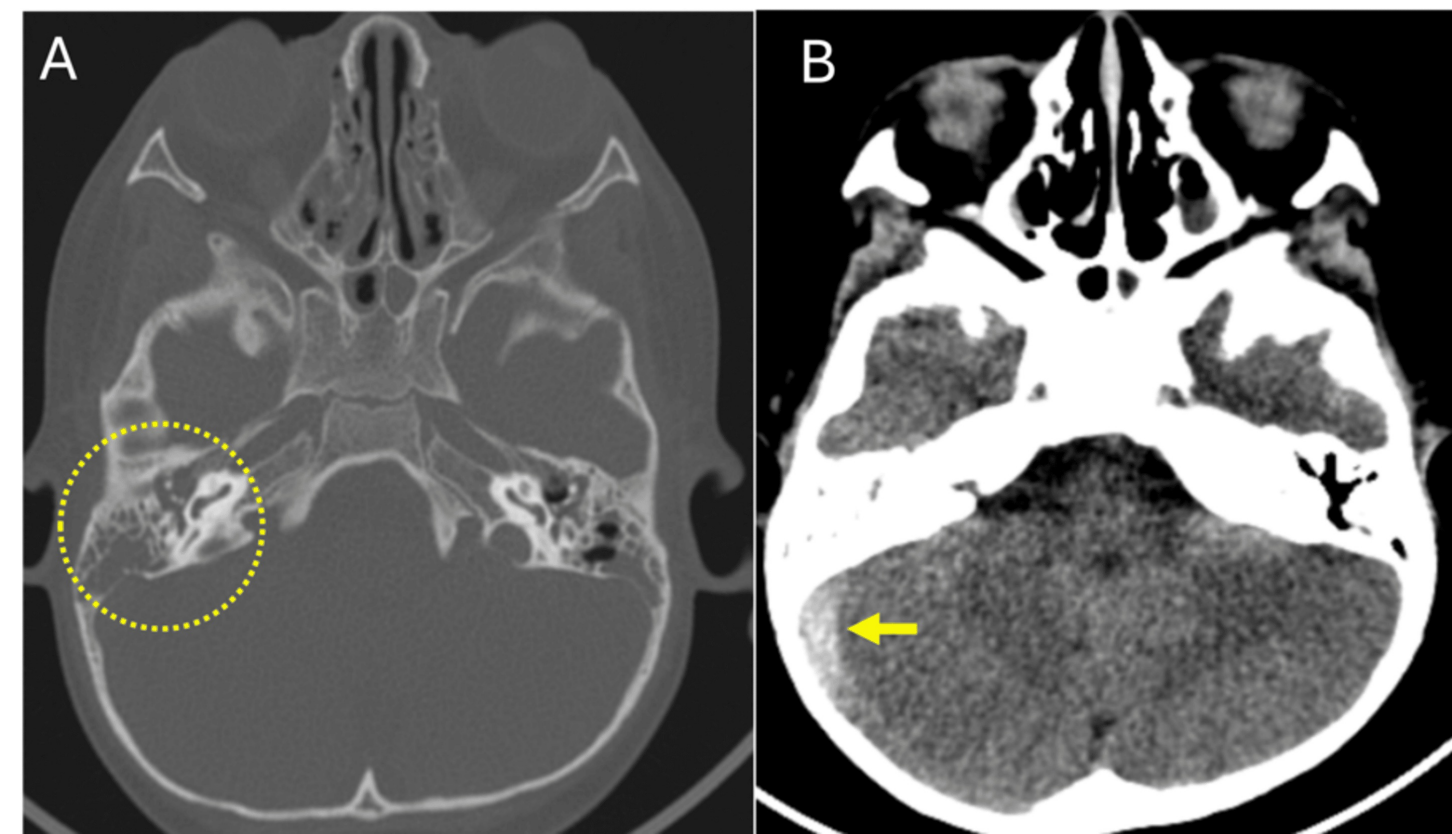
Cranial computed axial tomography. (A) The circle indicates soft tissue density occupying the right mastoid air cells and middle ear. (B) The arrow highlights increased amplitude of the transverse and sigmoid venous sinuses on the right side.

**Figure 2:**
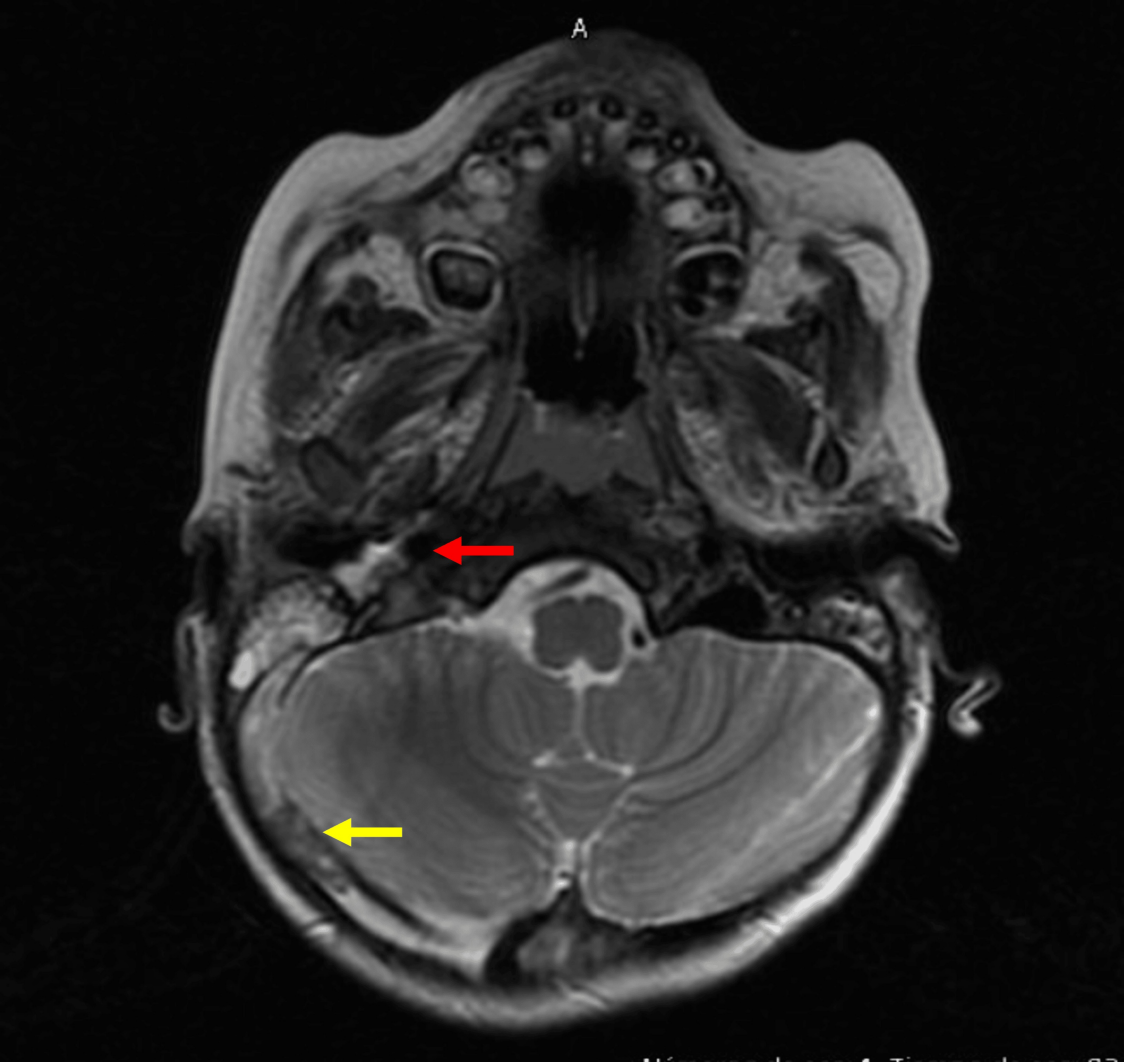
Magnetic resonance imaging: the red arrow indicates enhancement of the right jugular vein wall, suggesting thrombophlebitis; the yellow arrow shows thrombosis in the right transverse and sigmoid sinuses.

Cranial axial computed tomography revealed asymmetry of the transverse and sigmoid venous sinuses, with greater amplitude on the right side. There was also soft tissue density occupying the mastoid cells and both middle ears, as well as evidence of chronic inflammation in the right mastoid. The inner ears appeared normal. Secretions were present in the ethmoid, sphenoid, and maxillary sinuses (Figure 1). Management began with endovenous vancomycin at a dose of 210 mg every eight hours, ceftriaxone 530 mg b.i.d., subcutaneous low molecular-weight heparin (LMWH) 15 mg b.i.d.; nasal irrigations with saline solution and intranasal mometasone were initiated. Cerebral magnetic resonance imaging (MRI) also showed enhancement of the right jugular vein, a sign of thrombophlebitis well as enhancement of the soft tissues adjacent to the carotid sheath and absence of flow, which is consistent with thrombosis at the level of the transverse sinus, sigmoid sinus, and right jugular vein. Reactive lymph nodes in the right levels IIa and IIb (Figure 2).

**Figure 3:**
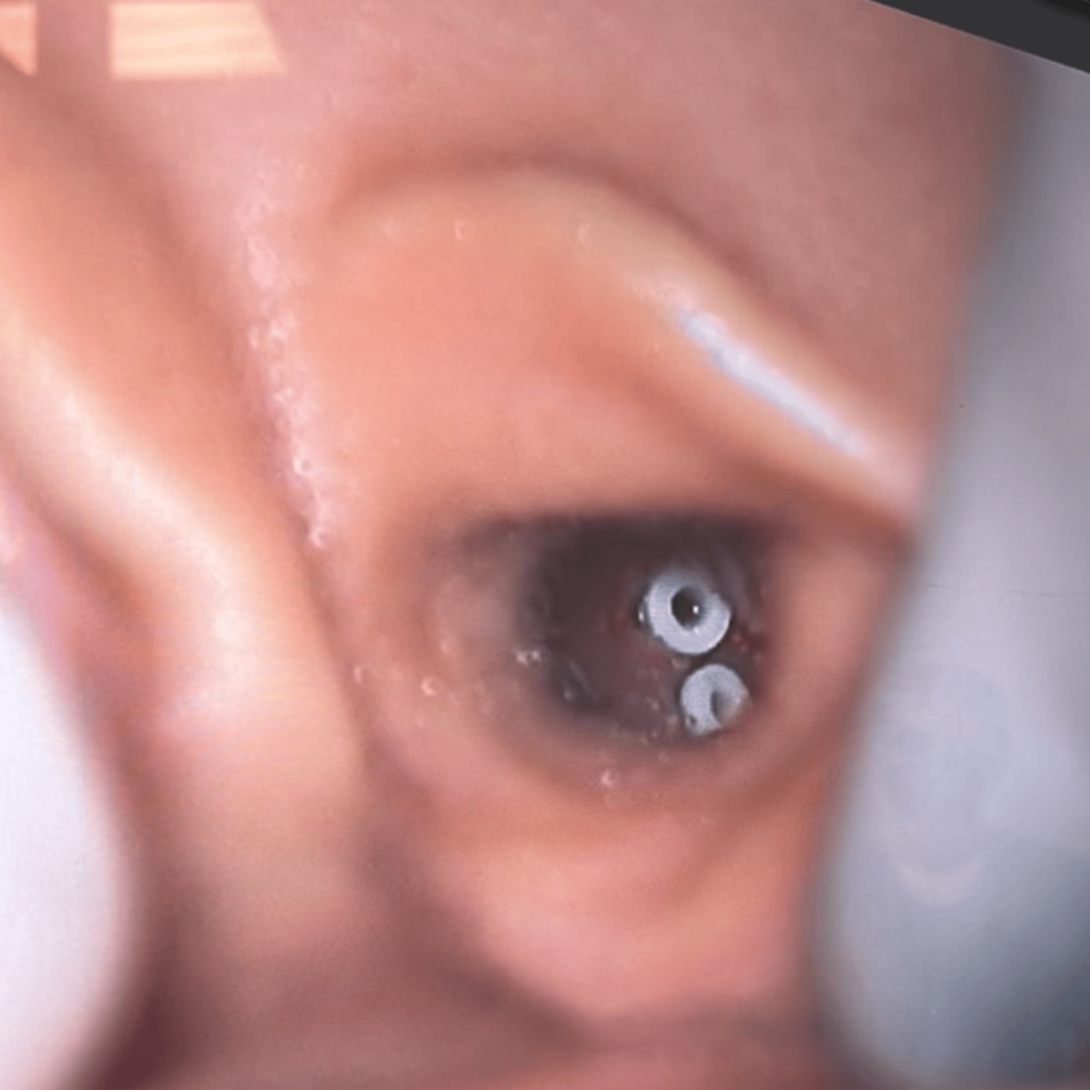
Tympanostomy tubes in the right ear.

**Figure 4:**
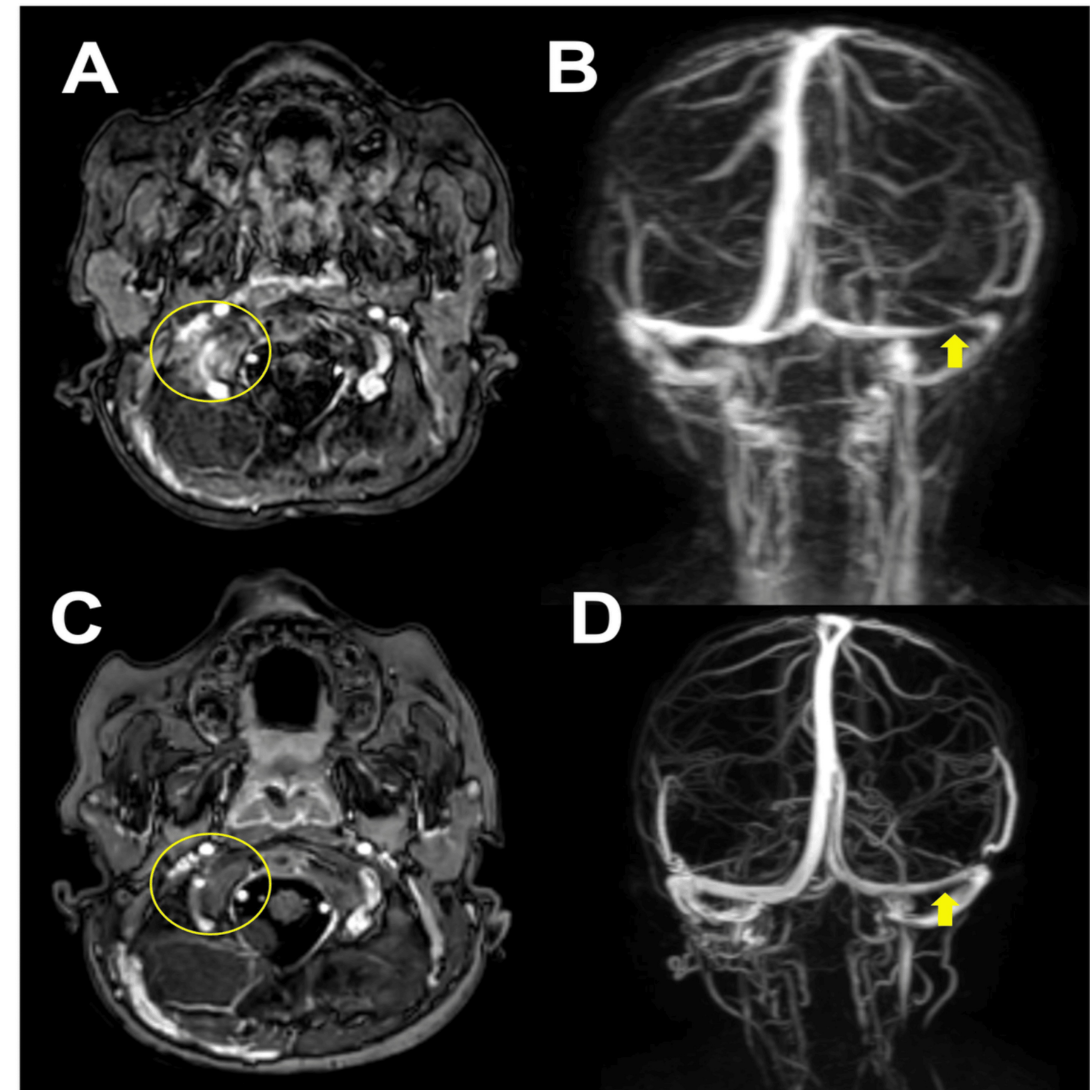
Magnetic resonance imaging and venogram. (A) The circle shows cerebellitis. (B) The arrow indicates partial venous thrombosis of the transverse sinus. (C, D) The circle and arrow show resolution of venous thrombosis.

After 36 hours, the crying, pain, and malaise disappeared. The patient was taken to the operating room for placement of two tympanostomy tubes in the inferior quadrants of the left ear and one tympanostomy tube in the inferior quadrant of the right ear to ensure adequate ventilation and drainage of the middle ear without observing commitment or additional risk of the procedure. No antibiotic drop treatment was left after the placement of the ventilation tubes (Figure 3). A sample sent for culture was positive for Streptococcus pyogenes at 100,000 CFU. Antineutrophil cytoplasmic antibodies (ANCA)-associated vasculitis and primary immunodeficiency were discarded as explanations, as the blood culture was negative. On the sixth day of hospitalization, the patient exhibited convergent strabismus of the right eye, associated with bilateral sixth cranial nerve paresis and ataxic gait; a new MRI was obtained, revealing persistent mastoiditis with altered bone intensity, indicative of osteomyelitis. The venous thrombosis appeared partial, and there was intra-axial intensity with enhancement adjacent to the cerebellum, suggesting cerebellitis (Figures 4A, 4B). Clinically, he had no history of seizures or altered consciousness.

**Figure 5:**
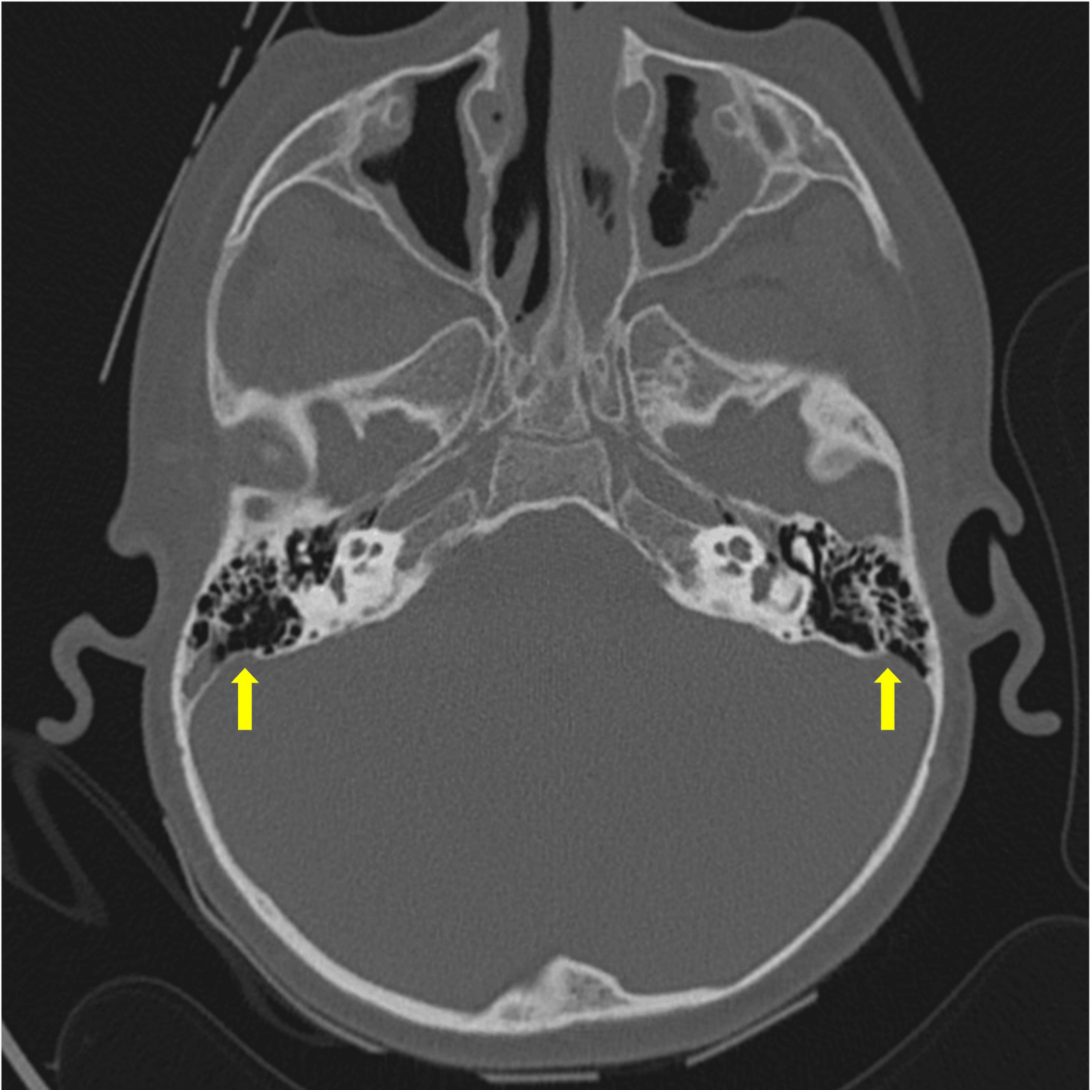
Temporal bone computed tomography: Both mastoid cavities are well-pneumatized, and the middle ear shows no signs of occupation.

After 16 days of the initial endovenous antibiotic treatment, he was discharged due to clinical improvement. Otoscopy showed only the presence of ventilation tubes without secretion or otorrhea, and inflammatory markers had normalized. He was instructed to continue home management with oral amoxicillin (90 mg/kg/day) for 21 days and anticoagulation for two weeks. At the two-month outpatient follow-up, no neurological changes were observed, and there was no evidence of tympanostomy tube extrusion. Three months later, a new MRI and venogram were performed, revealing collateral circulation and no recurrence of venous thrombosis (Figures 4C, 4B). Clinically, bilateral extrusion of the ventilation tubes was evidenced at the otoscopy. Six months after discharge, he underwent adenotonsillectomy and insertion of bilateral tympanostomy tubes for tonsillar hypertrophy grade III (Brodsky) and adenoid hypertrophy grade III, with data showing respiratory obstruction and a tympanogram showing Jerger curves type B. At the seventh month of postoperative follow-up, the patient had continued neurologically without compromise, with extruded tympanostomy tubes and no recurrence of AOM. The most recent ear CT, performed one year after hospitalization, showed data of bilateral chronic maxillary sinusitis and no changes in the density of the mastoid cavities (Figure 5).

## Discussion

In healthy children, AOM and AM are the most common predisposing conditions for cerebral venous sinus thrombosis (CVST) [6,7]. The preferred term *otogenic lateral sinus thrombosis *refers to the formation of thrombi in the sigmoid and/or transverse sinuses, with possible extension to the internal jugular vein [2]. Although its incidence is low, at least 0.67 per 100,000 children per year, its consequences can be fatal [7].

Thrombosis or thrombophlebitis can arise from the direct extension of infections in the middle ear or mastoid following mastoid bone erosion, which leads to necrosis extending to the intima and attracts fibrin, blood cells, and platelets, resulting in the formation of a mural thrombus or through hematogenous spread via emissary veins [8,9]. The thrombus can propagate in any direction, extending to the transverse sinus, superior sagittal sinus, internal jugular vein, or the cavernous sinus via the inferior or superior petrosal sinus [8]. The classic initial symptoms include pattern fever spikes above 39.4 °C, headache, and increased intracranial pressure; along with mastoiditis symptoms like earache, ear discharge, or Griesinger’s sign (edema over the posterior mastoid) [5,10]; diplopia and other neurological signs like abducens and facial nerve palsy, strabismus, seizures or ataxia, and even lethargy may be seen [7,11,12]. Performing neuroimaging is crucial in patients with high clinical suspicion. Contrast CT is the preferred first-line modality, as it is effective in ruling out brain tumors, abscesses, or arterial strokes. Findings like *dense triangle sign* are noted when thrombosis is present in the superior sagittal sinus, and *dense cord sign* when it is located in a cortical or deep vein, but these are rarely positive, occurring in only about 30% of patients with CVST [13-15].

Sensitivity in detecting sinus and venous thrombosis increases with contrast MRI; signs like the *empty delta sign*, which shows a filling defect in the center of the venous lumen with peripheral enhancement, support the diagnosis. MRI should be considered not only for diagnosis but also for the follow-up of these patients [1]. In the case of our patient, cranial CT and cerebral MRI were performed. The radiological findings included asymmetry of the transverse and sigmoid venous sinuses, with greater amplitude on the right side. Specific radiological signs such as the *dense triangle sign*, the *dense cord sign*, and the *empty delta sign* were not observed [15].

D-dimer has been investigated in several studies as a predictor of CVST, but it has consistently demonstrated low sensitivity and specificity [13,16]. In our patient, D-dimer levels were not measured at admission; however, on the second and seventh days, levels of 991 and 959 ng/mL were reported, respectively. The European Stroke Organization guidelines recommend measuring D-dimer before neuroimaging in patients with suspected CVST, except in those with isolated headache or symptoms lasting more than one week. However, the quality of evidence is low [13,17].

From a microbiological perspective, most cases of pediatric otogenic lateral venous sinus thrombosis have negative bacterial culture results. When positive, the most commonly isolated bacteria include Streptococcus pyogenes, Streptococcus pneumoniae, Staphylococcus aureus, Haemophilus influenzae, and Pseudomonas aeruginosa [1,18]. In the case of our patient, positive cultures were obtained for S. pyogenes. Studies report that positive cultures for Fusobacterium necrophorum are usually associated with a more aggressive course of the disease and osteomyelitis. For patients diagnosed with lateral sinus thrombosis, the literature recommends initiating broad-spectrum antibiotics, such as ceftriaxone, typically in combination with other agents like metronidazole or clindamycin, as early as possible. If a specific pathogen is subsequently identified, more specific antimicrobial agents should be substituted for the initial treatments [1].

Anticoagulation therapy and surgical treatment for otogenic lateral venous sinus thrombosis are still matters of debate [1]. In the literature, some studies suggest that patients with lateral sinus thrombosis require mastoidectomy to treat the underlying mastoid disease, as well as surgical drainage in cases of intracerebral complications such as brain abscess, according to the patient's condition [8]. The persistence of septicemia in a certain group of patients remains unexplained [19]. Clot management may involve anticoagulation, ligation of the jugular vein in the neck, and evacuation of the infected clot by opening the sinus.

Anticoagulant therapy is described as useful in restricting thrombus extension, promoting intracranial drainage, and limiting increases in intracranial pressure. It may be associated with complications such as bleeding, drug-drug interaction, thrombocytopenia, osteoporosis, and hemorrhagic skin necrosis [1].

Some authors suggest that anticoagulant therapy with low molecular-weight heparin should be initiated immediately after diagnosis and continued for two months or more in patients who fail to achieve recanalization or in those with high-risk thrombophilia [1]. Regarding surgical management, mastoidectomy can be performed in association with myringotomy, with or without tube placement. More aggressive options are not routinely recommended; ligation of the internal jugular vein is limited to cases of persistent sepsis or septic pulmonary embolism [1,20].

Our patient made a full recovery without any sequelae, following treatment that included the placement of two tympanostomy tubes in the right ear, a 21-day course of systemic antibiotics, and two weeks of anticoagulation with low-molecular-weight heparin. He did not require mastoidectomy later: radiological images showed complete resolution of thrombosis (Figure 5). A Cochrane database review concluded that the use of anticoagulation is safe and may lead to reduced mortality risk. In their treatment protocol, all children received anticoagulation therapy with either unfractionated heparin or low-molecular-weight heparin, provided there were no contraindications [12]. Based on our literature review and treatment protocol, we agree that anticoagulation could prevent thrombus propagation and improve the chances of sinus recanalization.

## Conclusions

This paper describes an intracranial complication of AM in a pediatric patient. A crucial aspect of this case is its possibly fatal outcome and dangerous sequelae, suggesting the importance of timely diagnosis and intervention. The mainstays of treatment are broad-spectrum antibiotics and anticoagulation therapy, which is necessary to block clot propagation and obtain recanalization, although it remains controversial.

Diagnosis and follow-up in this patient were made with MRI, the standard imaging modality to monitor the course of CVST resolution and assess for recanalization. Additional important points include inflammatory markers such as C-reactive protein and erythrocyte sedimentation rate, which were increased at the time of diagnosis and decreased with treatment; their role in the history of the disease is still unknown, and further studies are needed. In cases such as this, we recommend the placement of a double ventilation tube to ensure proper drainage and mastoid ventilation, alongside the administration of anticoagulants and broad-spectrum antibiotics. The role of corticosteroids in pediatric patients is still unclear and warrants further investigation through controlled studies. Our experience has shown that this approach leads to favorable outcomes, potentially reducing hospital stay and lowering the risk of hemorrhagic complications. Additionally, anticoagulant therapy has demonstrated general safety in young children when adequately monitored. Nevertheless, despite careful dosing and monitoring, the risk of complications must be balanced against the benefits of preventing thrombus propagation and improving clinical outcomes.
